# Attentional capture by Pavlovian reward-signalling distractors in visual search persists when rewards are removed

**DOI:** 10.1371/journal.pone.0226284

**Published:** 2019-12-12

**Authors:** Poppy Watson, Daniel Pearson, Steven B. Most, Jan Theeuwes, Reinout W. Wiers, Mike E. Le Pelley

**Affiliations:** 1 School of Psychology, UNSW, Sydney, Australia; 2 Department of Experimental and Applied Psychology, Vrije Universiteit, Amsterdam, The Netherlands; 3 Department of Psychology, University of Amsterdam, Amsterdam, The Netherlands; University of Sydney, AUSTRALIA

## Abstract

Existing research indicates that learning about the Pavlovian ‘signal value’ of stimuli can induce attentional biases: findings suggest that our attentional system prioritises detection of stimuli that have previously signalled availability of high reward. These findings potentially provide a human analogue of sign-tracking behaviour previously reported in studies of non-human animals. Here we examine a visual search task that has been developed to demonstrate the Pavlovian influence of reward on attention, in which the critical reward-signalling stimuli are never explicit targets of search. This procedure has previously yielded robust effects of reward on attention; however it remains unclear whether this pattern reflects a persistent and automatic bias in attentional capture based on prior experience of stimulus–reward pairings, or whether it results from participants strategically attending to reward-signalling distractors because they provide useful *information* about reward magnitude. To investigate this issue, in the current study participants initially completed a rewarded visual search task, in which colours of distractor stimuli signalled availability of high or low reward. Participants then completed a test phase in which rewards were no longer available, such that distractor colours no longer provided useful information on reward availability. Performance during the initial rewarded phase was impaired by the presence of a distractor signalling availability of high relative to low reward. Crucially, the magnitude of this reward-related distraction effect did not reduce in the subsequent unrewarded test phase. This suggests that participants’ experience of differences in reward value signalled by distractor stimuli in this task can induce persistent biases in the extent to which these stimuli involuntarily capture attention, even when they are entirely task-irrelevant.

## Introduction

Attention refers to the set of cognitive mechanisms that prioritize certain information for further analysis or action. A central line of research in the visual cognition literature has aimed to delineate the boundary conditions distinguishing times when we are in control of this prioritization process versus times when this prioritization occurs relatively automatically, driven by sensory information itself. This research points to a distinction between goal-directed and stimulus-driven attention [1, 2]. That is, we can control our attention to prioritize information relevant to our current task-goals (e.g., focussing on the road ahead while driving), but a particularly salient stimulus (e.g., a sudden, bright flashing light in the rear-view mirror) can *capture* our attention in a relatively automatic fashion, and receive priority regardless of our current goals.

**Fig 1:**
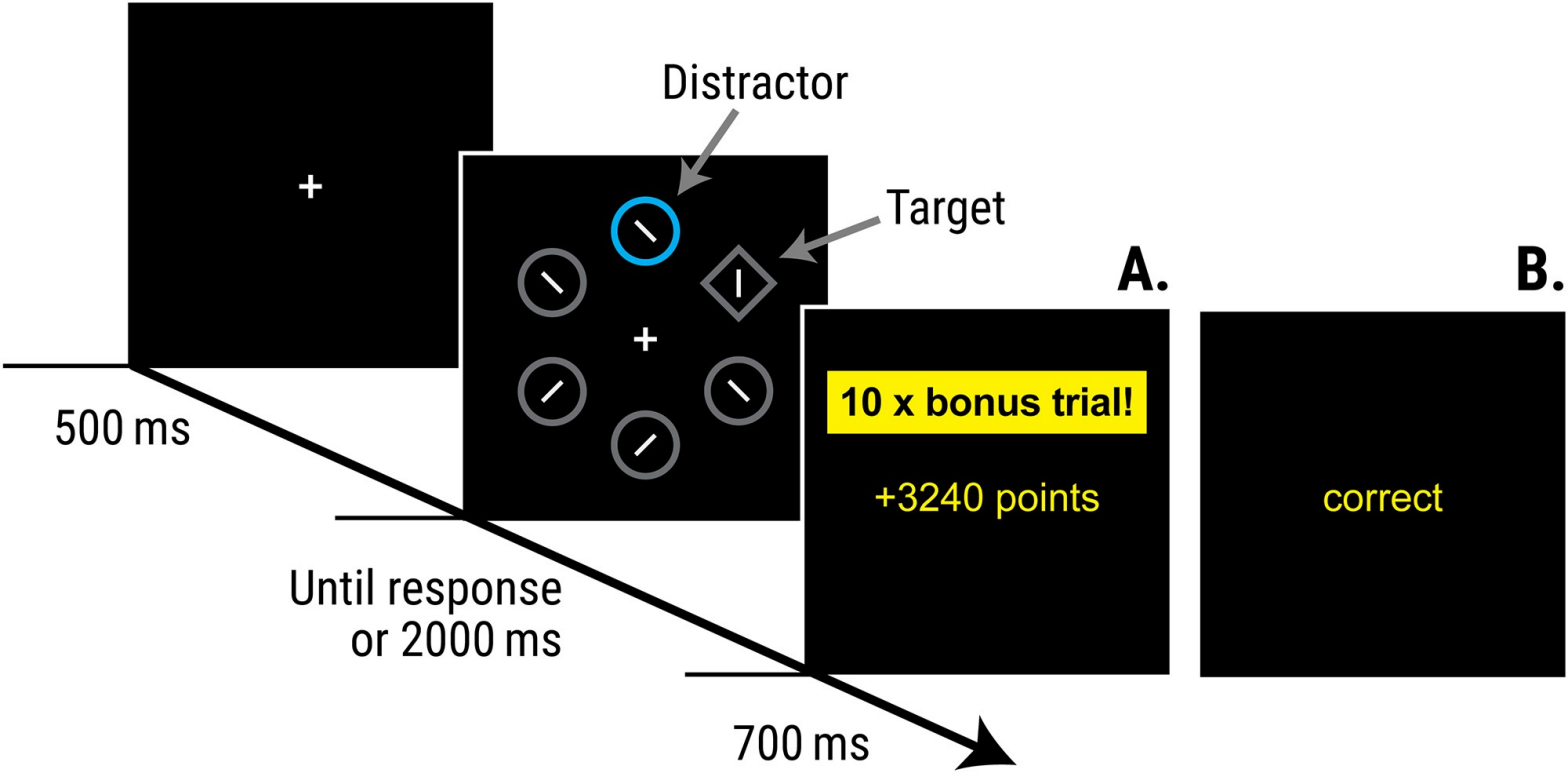
Trial structure of the VMAC task. Participants had to respond to the orientation of the line segment within the diamond (target); on the majority of trials, one of the circles in the display was a colour-singleton distractor. Only the feedback screens differed between phases of the procedure. During the reward phase **(A)** faster responses earned more points and the colour of the distractor signalled whether or not these points would be multiplied by 10 (bonus trials, as depicted here). During the unrewarded test phase **(B)** feedback simply informed participants whether or not they had responded correctly.

A recent body of research suggests that the likelihood that a stimulus will capture our attention is critically influenced by our prior experiences with that stimulus, and in particular by what we have learned about how that stimulus relates to motivationally significant events, such as rewards and punishments [3–6]. Several procedures have now been developed for studying the influence of rewards on capture of visual attention [7–12]. Here we focus on a version of the additional singleton task [13] which was adapted by Le Pelley et al. [10]. On each trial of this task, participants searched for and responded to a diamond target among circles; the faster their correct responses, the larger the reward they earned (see Fig 1). On most trials, one of the circles was coloured either blue or orange; all other shapes were grey. We refer to the colour-singleton circle as the *distractor* to distinguish it from the other (grey) circles in the display, which we refer to as non-salient non-targets. If the distractor circle appeared in the high-reward colour (say blue) this signalled that the current trial was a bonus trial on which reward would be multiplied by a factor of 10; if the distractor was in the low-reward colour (orange in this case) the current trial was not a bonus trial. Notably, while the distractor signalled reward magnitude, it was not the target that participants responded to in order to receive that reward. The optimal strategy in this task is therefore to ignore the distractors and respond to the target as rapidly as possible, since this would yield highest earnings. Nevertheless, responses to the target were significantly slower (but no more accurate) for trials featuring a distractor that signalled availability of high reward, than for trials with a distractor that signalled low reward. The implication is that the high-reward distractor was more likely to capture participants’ attention, interfering with (and slowing) search for the target–even though this behaviour was counterproductive because it meant participants earned less on high-reward trials than would otherwise have been the case. In other words, these findings suggest that learning about the value of the reward that is signalled by a stimulus can change the likelihood that it captures attention in future [14–19], a phenomenon termed *value-modulated attentional capture* (VMAC).

This procedure for studying VMAC is notable because the critical reward-related stimuli indicate the magnitude of reward available on each trial, but participants are not required to respond to or attend to these stimuli in order to receive that reward: in the language of learning theory, the distractor colours are Pavlovian signals of reward (compare with other VMAC procedures in which reward-related stimuli are initially trained as targets of search and hence have an instrumental relationship with reward [7, 20]). In this regard, demonstrations of VMAC using this Pavlovian task bear relation to studies of *sign tracking* in nonhuman animals [21, 22]. Sign tracking refers to the tendency of some animals to learn to approach a Pavlovian signal of reward rather than the location at which reward is actually delivered: for example, if insertion of a lever signals delivery of food to a magazine, some rats—termed sign trackers—come to approach and interact with (touch, gnaw etc) the lever when it is inserted, rather than learning to approach the food magazine [e.g., 23, 24]. The parallel with the VMAC procedure described is clear: in both cases the effect is that a signal of reward comes to exert control over behaviour, which manifests either in overt approach (in the case of sign tracking in animals) or in an attentional bias (in VMAC). Notably, sign-tracking traits have been associated with behavioural signs of executive control deficits, and have been argued to constitute a cognitive risk marker for compulsive or otherwise maladaptive cue-driven behaviours, particularly with regard to addiction [23, 25, 26]. And consistent with the idea of a parallel between VMAC and sign tracking, human studies using the VMAC procedure described above have demonstrated that the magnitude of the attentional bias to Pavlovian signals of high reward (versus low reward) is associated with levels of compulsive and addiction-related symptoms [14, 27–29].

In line with research on sign tracking, we have characterised VMAC as reflecting a relatively automatic influence of reward-related incentive salience on attentional priority–that is, pairings of a distractor colour with high reward render that colour more salient and hence more likely to capture visual attention in future, regardless of the participants’ current task-goals. However, an alternative interpretation is possible. Although participants are never required to respond to the critical distractor stimuli in this task, these stimuli do provide information on whether responding to the target will produce high or low reward: that is, they have *informational value* [30]. This raises the possibility that participants may try to use the reward-related stimuli strategically, to identify which trials will yield a large reward. This is a poor strategy to use since it will result in slower responses, and hence lower rewards, than if participants ignored the distractors. Yet it remains possible that the impaired performance on high-reward trials is a consequence of strategic attentional selection of the high-reward distractor (based on its information value) rather than involuntary capture (based on its reward value) [11, 31, 32].

This issue formed the focus of the current study, in which we asked whether the ‘sign tracking’ VMAC procedure could indeed induce a change in the likelihood that reward-related distractors will capture attention, or whether attention to reward-related distractors in this procedure is purely strategic and goal-directed, based on current information value. Following training on the VMAC task as described earlier, participants completed a final test phase in which they were explicitly informed that no further rewards were available. Consequently, the critical reward-related distractors no longer carried useful information regarding reward magnitude during this unrewarded test phase—participants already knew that no reward would be available on each trial—and so there was no strategic reason to attend to them. The unrewarded test phase used in the current task was relatively short, but sufficient for testing the potential role of strategic processes in attentional bias to reward signals: if any such attentional bias that emerged during the reward phase reflected strategic orienting based on current informational value, then it should vanish immediately when participants become aware that rewards are available (and hence distractors no longer provide information). The implication is that any attentional bias towards the high-reward distractor that persisted during the unrewarded test phase would demonstrate that prior training caused changes in capture, i.e., that pairings with reward can induce a persistent change in the attentional priority of a stimulus in a way that goes beyond strategic allocation of attention based on current informational value [5, 31].

## Method

### Participants and Apparatus

This study was approved by the UNSW Sydney Human Research Ethics Advisory Panel (Psychology). Participants were 272 undergraduate students who completed the study as part of a practical class, in groups of approximately 40, using individual computers with 23-in. monitors (1920 × 1080 resolution, 60 Hz refresh) at a viewing distance of ~60 cm. Stimulus presentation was controlled by MATLAB using Psychophysics Toolbox extensions [33–35]. Experiment scripts are available at https://osf.io/mr8px. Nine participants were excluded on the basis of low accuracy or excessive anticipations/time-outs (see Results). The remaining 263 participants (190 females) had a mean age of 20.8 (*SEM* = 0.24) years.

### Stimuli and design

The VMAC task (Fig 1) was based on that reported in [10]. Each trial began with a central fixation cross, followed after 500 ms by the search display: a set of six shapes (size: 2.3° × 2.3° visual angle) arranged evenly around an imaginary ring (diameter 10.1°). Five of these shapes were circles, each containing a white line tilted 45° randomly to the left or right. One shape (the target) was a diamond containing a line oriented (randomly) horizontally or vertically. On most trials, one of the circles (the distractor) was coloured; all other shapes were grey. For half of participants, distractor colours were blue and orange; for remaining participants, distractors were green and pink. Assignment of blue/pink and orange/green to the roles of *high-reward* and *low-reward* colours was counterbalanced across participants within each of these groups. Target and distractor location were randomly determined on each trial.

Participants’ task was to report the orientation of the line within the diamond target as quickly as possible—by pressing either ‘C’ (horizontal) or ‘M’ (vertical)—with faster responses earning more points. Each block comprised 24 trials: 10 with a distractor in the high-reward colour, 10 with a distractor in the low-reward colour, and 4 distractor-absent trials (in which all shapes were grey). For correct responses, on low-reward-distractor and distractor-absent trials, participants were awarded 0.1 points for every ms that their response time (RT) was below 1000 ms (so an RT of 600 ms would earn 40 points). Trials in which the display contained a high-value distractor were labelled as bonus trials, and points were multiplied by 10 (so an RT of 600 ms would earn 400 points). Correct responses with RT > 1000 ms earned no points, and errors resulted in loss of the points that otherwise would have been won. The search display remained on-screen until a response was registered or the trial timed-out (after 2000 ms). A feedback screen was then presented for 700 ms. If the trial had timed-out, feedback was: “Too slow. Please try to respond faster”. Otherwise, during the *reward phase* of the task (see Procedure), if the response was correct, feedback showed the number of points won (e.g., “+350 points”); if the response was incorrect, feedback showed “ERROR” and the number of points lost (e.g., “ERROR: Lose 350 points”). On high-reward trials feedback was accompanied by a box labelled ‘10 × bonus trial!’. During the unrewarded test phase, only accuracy feedback (“correct” or “incorrect”) was presented and neither points information nor the bonus box appeared. Inter-trial interval was 1200 ms.

### Procedure

Participants were informed that their aim was to earn as many points as possible. Due to the classroom setting our ethical approval meant we could not give monetary bonuses, so as an alternative source of motivation points were used to unlock ‘medals’. For every 10,000 points that participants earned, they unlocked a new medal (in the order bronze, silver, gold, platinum, diamond, and elite). Based on mean RTs from our previous work [10], this would mean that the best-performing ~10% of participants would unlock the ‘elite’ medal.

Instructions prior to the initial rewarded phase informed participants (1) that faster (correct) responses would earn more points, (2) that when a circle in the high-reward colour appeared in the search display it would be a bonus trial on which points were multiplied by 10, and (3) that when a circle in the low-reward colour appeared it would not be bonus trial. Check-questions verified that participants understood these instructions: participants were required to respond correctly to these questions before they could continue. Participants then completed twelve blocks of 24 trials (288 trials total) in the reward phase of the task, taking a break after every two blocks during which they were shown their total number of points, and an animation presented any medals unlocked since the previous break.

On completing the reward phase, on-screen instructions stated that: “*FROM NOW ON*, *THERE ARE NO MORE POINTS AVAILABLE*! *This means that you will not win (or lose) any points for the rest of the game*. *Nevertheless*, *you should carry on responding to the orientation of the line within the diamond as quickly and accurately as possible*.*”* Again check-questions were used to verify participants had understood these instructions. Participants then completed two blocks of 24 trials (48 trials total) in the unrewarded phase.

## Results

All raw data are available at https://osf.io/mr8px. Following our previous protocol [10] we discarded data from the first two trials after each break, time-outs (0.4% of all trials), and trials with RTs below 150 ms (anticipations: 0.6% of all trials). Analysis of RTs used correct responses only. Since we were primarily interested in the effect on performance of transitioning from the reward phase to the unrewarded test phase, analyses focused on the data from the final two blocks of the reward phase and the two blocks of the unrewarded test phase. As a conservative measure, we excluded participants who had more than 25% of their data discarded from these critical blocks of the reward or unrewarded phase (due to anticipations or time-outs), and any participant who scored below 50% accuracy in the visual search task. Data from nine participants were removed on this basis; we note that removal of these participants had no effect on the pattern of significant findings in this study.

**Fig 2:**
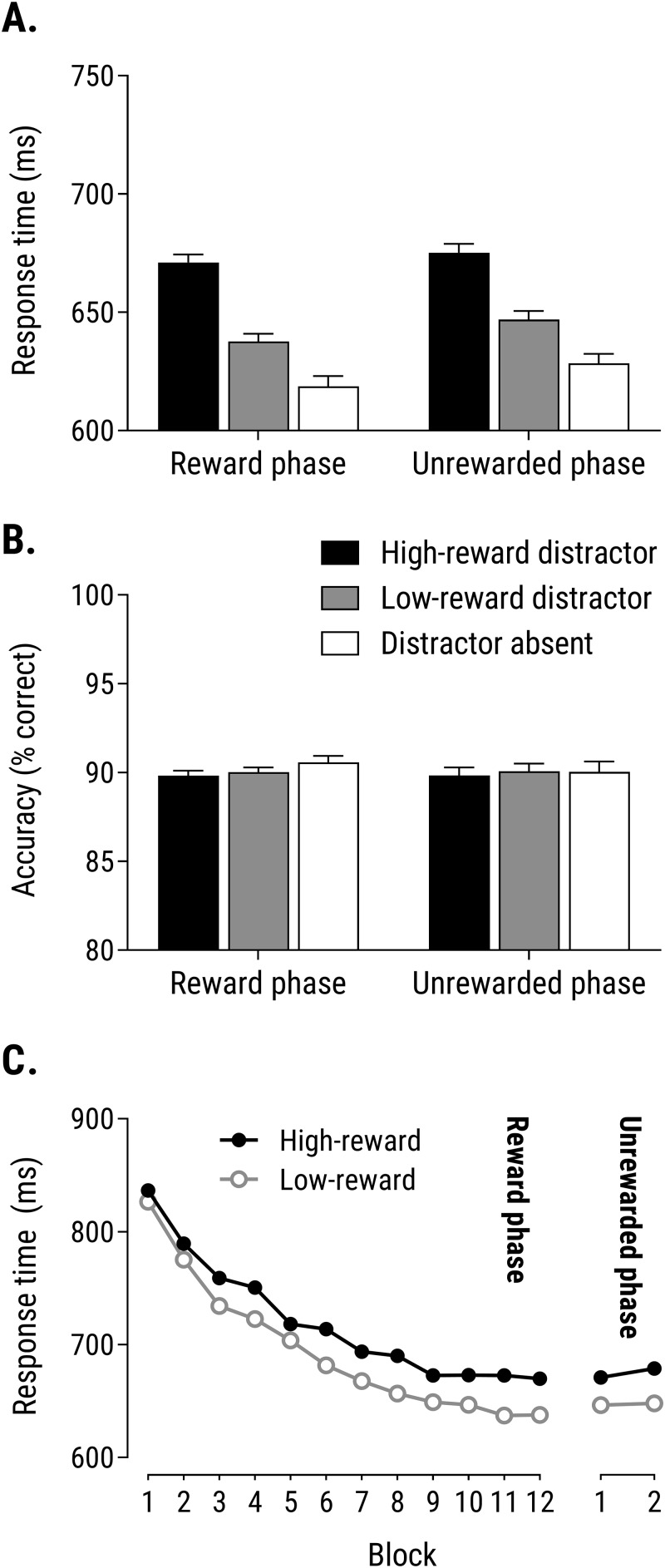
**(A)** Mean response time and **(B)** accuracy from the final two blocks of the reward phase and the two blocks of the unrewarded test phase as a function of distractor type (high reward, low reward and distractor absent). Error bars represent within-subject standard error of the mean [38]. **(C)** Mean response time for trials with a high- or low-reward distractor across the 12 blocks of the reward phase (left-hand section), and the 2 blocks of the unrewarded phase (right-hand section).

Fig 2A shows the data of main interest: that is, RTs from the reward phase and unrewarded test phase, for trials in which the search display contained a high-reward distractor, a low-reward distractor, or no colour-singleton distractor (distractor-absent trials). The pattern was similar in both phases, with slower responses on trials with a colour-singleton distractor versus distractor-absent trials, and (more importantly) slower responses on trials with a high-reward distractor versus a low-reward distractor. A 3 (distractor type: high-reward, low-reward, or distractor-absent) × 2 (phase: rewarded versus unrewarded) repeated measures ANOVA revealed a significant effect of distractor type, *F*(2,524) = 102.4, *p* < .001, η_*p*_^2^ = 0.28, but no main effect of phase, *F*(1,262) = 3.26, *p* = .072, η_*p*_^2^ = 0.01, and no interaction *F* < 1. We followed-up with a 2×2 ANOVA analysis restricted to the high-reward and low-reward distractor trials of each phase, since this contrast isolates the effect of reward magnitude (specifically) on behaviour. This analysis revealed a significant main effect of distractor type, *F*(1,262) = 85.6, *p* < .001, η_*p*_^2^ = 0.25, but no main effect of phase, *F*(1,262) = 2.39, *p* = .12, η_*p*_^2^ = 0.009, and no interaction, *F* < 1. Thus the high-reward distractor slowed responses relative to the low-reward distractor, and the size of this effect of reward on RT did not differ significantly in the rewarded and unrewarded phases. Planned t-tests revealed that the high-versus-low RT difference was significant in both the reward phase, *t*(262) = 8.05, *p* < .001, *d*_*z*_ = 0.50, and the unrewarded phase, *t*(262) = 6.54, *p* < .001, *d*_*z*_ = 0.40. We conducted a Bayesian t-test using JASP [36], with the default Cauchy prior, to compare the magnitude of the VMAC effect between the two phases. This revealed substantial support [37] for the null hypothesis of no difference between the two phases versus the two-tailed alternative hypothesis of a difference in VMAC effect between the two phases, *BF*_01_ = 8.95, and substantial support for the null over the one-tailed alternative hypothesis that the VMAC effect would be greater in the reward phase than in the unrewarded phase, *BF*_01_ = 5.36.

Fig 2B shows that mean accuracy was high in both phases for all distractor types. Analysis of accuracy data via a 3 (distractor type: high, low, absent) × 2 (phase) ANOVA found no significant main effects or interaction, all *F*s < 1. Bayesian t-tests were used to compare accuracy for high- and low-reward distractor trials in the reward and unrewarded phases. These tests yielded strong support for the null hypothesis of no difference in accuracy versus the two-tailed alternative hypothesis in both phases, *BF*_01_ = 10.3 and 13.3 respectively, and even stronger support for the null versus the one-tailed alternative (based on the possibility of a speed–accuracy trade-off) of greater accuracy on high-reward trials, *BF*_01_ = 25.1 and 19.4 respectively. Thus the critical slowing of responses when the display contained a high-reward versus a low-reward distractor was *not* accompanied by an increase in response accuracy, indicating that the effect of reward magnitude on RT did not reflect a speed–accuracy trade-off.

We also analysed how the difference in RT for trials with a high-reward versus low-reward distractor—our critical measure of the influence of reward on attention—evolved over the course of the initial rewarded phase (see Fig 2C, left-hand section). A 2 (distractor type: high- vs low-reward) × 12 (block of the rewarded phase) ANOVA revealed a main effect of distractor type, *F*(1,262) = 120.1, *p* < .001, η_*p*_^2^ = 0.31, with slower responses overall on trials with a high-reward distractor than trials with a low-reward distractor, and a main effect of block, *F*(11,2882) = 168.0, *p* < .001, η_*p*_^2^ = 0.39, with RTs generally becoming faster as the experiment progressed. There was also a significant interaction, *F*(11,2882) = 2.14, *p* = .015, η_*p*_^2^ = 0.08, that showed a significant linear trend, *F*(1,262) = 10.9, *p* = .001, η_*p*_^2^ = 0.04, with the RT difference between high- and low-reward trials increasing as participants gained more experience of the colour–reward relationships over the course of the rewarded phase.

Finally, we conducted a finer-grained analysis of RT data from the critical unrewarded phase. The right-hand section of Fig 2C shows RT for high- and low-reward distractor trials in the two 24-trial blocks of the unrewarded phase. A 2 (distractor type: high- vs low-reward) × 2 (block) ANOVA revealed a main effect of distractor type, *F*(1,262) = 41.0, *p* < .001, η_*p*_^2^ = 0.14, with slower responses on trials with a high-reward distractor than trials with a low-reward distractor. The main effect of block was not significant, *F*(1,262) = 1.15, *p* = .284, η_*p*_^2^ = 0.004, and neither was the interaction, *F* < 1. That is, the influence of distractor–reward relationships on RT (i.e., the VMAC effect) did not differ significantly between the two blocks of the unrewarded phase. Paired t-tests revealed that the VMAC effect (difference in RT between high- and low-reward distractor trials) was significant in each of blocks 1 and 2 considered separately, smaller *t*(262) = 3.95, *p* < .001, *d*_*z*_ > .24. A Bayesian t-test revealed strong support for the null hypothesis of no difference in the size of the VMAC effect in blocks 1 and 2 versus the two-tailed alternative hypothesis of a difference, *BF*_01_ = 10.6, and even stronger support for the null over the one-tailed hypothesis that the VMAC effect would be smaller in block 2 than in block 1, *BF*_01_ = 24.7.

## Discussion

In a replication of previous findings [10], during the rewarded phase of the current experiment, participants were slower (but not more accurate) to respond to the target on trials in which the search display contained a distractor signalling availability of high reward compared to low reward. This implies that participants were more likely to attend to the high-reward distractor than the low-reward distractor, thus interfering with search for the target. This influence of the distractor on attention (and performance) was counterproductive, since response times influenced the points that were earned during the rewarded phase–by responding more slowly (but no more accurately) on trials in which the highest rewards were available, participants lost out disproportionately. Critically, the current study extends previous work by demonstrating that the influence of the distractor’s reward history produced by this procedure persisted during a subsequent, unrewarded phase in which participants were aware that no more rewards were available, rendering the distractors entirely task-irrelevant since they no longer provided information on the magnitude of reward available on each trial (i.e., they had zero informational value). The implication is that the attentional bias towards high-reward distractors—the VMAC effect—was not (solely) a consequence of strategic orienting based on the information provided by these distractors, as it continued even when they no longer provided any information. Of course, updating of strategic control of attention may not be absolutely immediate: the mere instruction that rewards would be removed (prior to the unrewarded test phase) may be insufficient to encourage participants to update their goal-directed attentional control settings. For example, participants may not believe this instruction. Notably, however, Bayesian analysis indicated that the magnitude of the VMAC effect did not reduce over the two blocks of the unrewarded test phase: even direct experience that rewards were no longer available in the first block of this test phase (confirming the truth of what participants had previously been told) had no measurable impact on the attentional bias that was observed. That is, even though participants had been told, and had experienced, that distractors no longer provided reward information, the VMAC effect was not diminished in the second block of the unrewarded phase. The finding of a VMAC effect even when participants had had ample opportunity to update strategic control suggests that the effect was not goal-directed. Instead, our findings indicate that participants’ experience during the training phase led to a persistent and automatic attentional bias towards the stimulus that had previously signalled high reward, such that this stimulus became more likely to capture attention in future even though this was contrary to participants’ task goals. Indeed, the transition from the rewarded phase to the unrewarded phase had no significant impact on the extent to which the previously-reward-signalling distractors influenced performance, with a Bayesian analysis providing substantial support for the null hypothesis of no difference in the size of the VMAC effect before and after the transition. This suggests that strategic orienting on the basis of current informational value made little (if any) contribution to the response-time difference observed at the end of the rewarded phase.

The current findings bear some similarity to previous work on the influence of Pavlovian reward-signalling stimuli by Mine and Saiki [39]. In that study, participants initially performed a rewarded *flanker task* in which the colour of non-target flanker stimuli signalled whether a correct response to a target stimulus would yield high or low reward. After training on this rewarded flanker task, participants moved to an unrewarded visual search task, broadly similar to that used in the current study. Responses to the target in this test phase were slower when the display contained a distractor in a colour that had previously signalled high reward versus low reward. In line with the current findings, these results are consistent with the idea that establishing stimuli as Pavlovian signals of reward can induce persistent changes in the extent to which they capture attention in future, independent of their current informational value. Notably, however, half of the trials in Mine and Saiki’s flanker task were ‘congruent’ trials in which the correct response to the target was the same as the response associated with the distractor letters; hence on these congruent trials a response made to the distractor (rather than to the target) would also yield reward. Consequently, distractor stimuli were not merely reward *signals* in this task, since participants could also (sometimes) receive reward for responding to these stimuli. By contrast, in the current study the distractor stimuli did not afford any response (the line segment in the distractor was never vertical or horizontal: see Fig 1), and so these stimuli only ever functioned as signals of reward–and this allows us to isolate the effect of reward to the signalling contingency more precisely. Moreover, Mine and Saiki did not observe a significant influence of the reward-signalling status of the distractor colours on performance during the initial, rewarded flanker task. As such they were unable to observe the emergence of the reward-related attentional bias during the training phase; it was revealed only in the test phase, once fully-formed. By contrast, we observed a clear effect of the reward-signalling status of the distractor during the initial rewarded phase of the task, in that a high-reward distractor significantly impaired performance relative to a low-reward distractor in training just as in test (see Fig 2A). As such the procedure used here is perhaps better-suited for studying the development of attentional biases as a result of experience of differences in the ‘signal value’ of stimuli.

In demonstrating that previous experience can influence the ongoing and automatic attentional prioritisation of reward-signalling stimuli, the current findings underline the parallel between the VMAC effect and previous demonstrations of sign tracking in nonhuman animals [21–24, 40]. Rodent studies of sign tracking have provided important insight into the psychology and neurobiology of reward pathways that are thought to be implicated in maladaptive cue-driven behaviours such as those associated with compulsivity and addiction. To the extent that it provides a human analogue of sign tracking, the VMAC procedure used here may constitute an important tool for translation of these prior findings into human research, and preliminary work along these lines has yielded promising findings [14, 27–29].

While our findings provide an analogue of sign-tracking behaviour in attentional capture, it remains an open question whether the learning process that yields this behaviour is Pavlovian or instrumental in nature. One possibility is that experience of pairings of a signal (here a distractor colour) with high reward leads to that signal acquiring *incentive salience* through a process of Pavlovian conditioning [40], such that the signal becomes desirable in its own right, and able to motivate approach behaviour–which manifests here as a bias in automatic attentional capture that persists when rewards are subsequently removed. An alternative possibility is that the root of the automatic attentional bias to reward signals is instrumental in nature, based on reinforcement associated with information seeking. On this account, participants learn that looking at the high-reward distractor provides useful information (that a high reward is available), and this gain in information provides reinforcement for the conditioned, instrumental response of attending to the high-reward distractor. To clarify, our findings indicate that, by the end of the reward phase, the attentional bias towards the high-reward distractor is automatic; it does not reflect goal-directed, strategic orienting on the basis of the current informational value of distractors. However, it remains possible that instrumental conditioning based on the reinforcement provided by informational value results in an *automatic* attentional bias towards the high-reward distractor that persists into the unrewarded phase. Indeed, it is even possible that strategic orienting based on informational value may have contributed to an initial bias in attention to the high-reward distractor earlier on in the rewarded phase, but that over many trials of training in the rewarded phase this behaviour becomes automatic, stamped in as an ‘attentional habit’ [3]. This possibility would be consistent with recent theorising that, with experience, attentional biases based on selection history can transition from controlled, top-down effects to automatic biases that exert an enduring effect on behaviour [41].

To summarise, distractor colours were Pavlovian signals of reward magnitude in our task; however we cannot know for sure if the learning process that produced the observed bias in automatic attentional capture by these reward signals was itself Pavlovian (learning that a stimulus signals high reward causes an increase in its incentive salience), or if instead it was instrumental (attending to a signal of high reward is reinforced by the information that this signal provides). Functionally, the effect on behaviour—‘attentional sign tracking’—would be the same in both cases, and hence the distinction is largely academic. Indeed, these alternatives may be inextricably linked, given that—by definition—a Pavlovian signal of high reward provides information that a high reward is available. That said, we note that previous work in animals has attempted to distinguish contributions of Pavlovian and instrumental conditioning to learning about reward signals [42]; it remains an open question as to whether analogous approaches could be used in studies of human reward learning.

Up to this point, we have described our findings as indicating an influence of reward on attention. However, we should note a caveat here. Consistent with some of our previous research (Le Pelley et al., 2015, Experiments 1 and 2), in the current task erroneous responses resulted in loss of the amount that would otherwise have been won on that trial. This was done in order to discourage participants from anticipating the correct response on each trial, which would result in very high rewards when they guessed correctly (since RT would be very short), and no losses when they were incorrect. However, we note that this manipulation meant that the high-reward distractor also signalled the possibility of a greater *loss* than the low-reward distractor. Hence we cannot tell for certain whether the critical factor influencing attention in this task was learning about reward or learning about loss/punishment, and so a more agnostic view would see this study as demonstrating an influence on attentional capture of learning about ‘value’ rather than reward per se. We note that accuracy was generally high in this task (~90% correct responses), so participants had considerably more exposure to reward than punishment contingencies; we also note that previous, related work has used procedures in which losses never occur, and effects of ‘pure’ reward on attention were observed that mirror those from the reward phase of the current procedure [e.g., 14, 15–17, 27–29]. As such it seems likely that reward exerted a critical effect on attention in this study. Nevertheless, future work could use a reward-only procedure in conjunction with the current task design in order to confirm that attentional effects of reward learning (specifically) rather than value learning (more generally) persist to influence attentional capture when rewards are removed.

There were some limitations to the current study. First, the classroom setting meant that there were constraints on the duration of the task. We provided considerable experience of the colour–reward contingencies in the rewarded phase (288 trials), to ensure that participants encoded these relationships with sufficient strength that they were clearly manifest in behaviour. However, this meant that the unrewarded phase had to be relatively short (48 trials). As noted earlier, this short test phase was sufficient and appropriate for the research question addressed here—investigating the immediate effect on performance of a change from rewarded to unrewarded conditions—but meant that we could not track changes in the VMAC effect over the course an extended unrewarded phase (during which time colour–reward associations would presumably extinguish). Future studies using a longer unrewarded phase could further examine the persistence and dynamics of the underlying processes [cf. 20]. Another limitation is that rewards were entirely symbolic in this task: participants earned points and virtual ‘medals’, but these had no tangible value in terms of standard primary or secondary reinforcers (e.g., food or money). Nevertheless, we observed a sizeable effect of distractor-type on performance, indicating that the points were inherently motivating to participants [see also 28]. Finally, the reward-related distractors used in the current task were colour-singletons, and hence might be expected to capture attention on the basis of their physical salience [13] regardless of their relationship with reward. The current study clearly demonstrates that reward can *change* the likelihood that distractors will capture attention, but was unable to test whether reward can *cause* distractors to capture attention when they otherwise would not have done so. This is the fundamental distinction between value-*modulated* capture, and value-*driven* capture which refers to cases in which physically non-salient stimuli come to capture attention by virtue of their relationship with reward [5, 7, 17]. Previous research has used a similar visual search procedure with reward-related distractors that were not physically salient [15], and future studies could pursue whether this procedure does indeed *drive* attentional capture by these distractors by incorporating an unrewarded test phase as in the current study.

In summary, the current study investigated the nature of the attentional bias to reward-related stimuli that results from a visual search procedure in which these stimuli signal the magnitude of reward available on each trial, but are never the targets that participants must respond to in order to earn that reward. We found that performance in this visual search task was impaired by the presence of a distractor that signalled the availability of high relative to low reward. Crucially, the magnitude of this reward-related distraction effect did not reduce in an unrewarded phase where participants were informed that points were no longer available. This suggests that this task can produce a persistent and automatic bias in the attentional priority of the reward-signalling distractors that is not a consequence of their current informational value, but instead reflects their prior history of pairings with reward. The similarities between this behaviour and sign tracking in animals are striking and future studies could use the VMAC paradigm presented here to probe this parallel in more detail.
